# More pieces to a huge puzzle: Two new *Escovopsis* species from fungus gardens of attine ants

**DOI:** 10.3897/mycokeys.46.30951

**Published:** 2019-02-18

**Authors:** Quimi Vidaurre Montoya, Maria Jesus Sutta Martiarena, Danilo Augusto Polezel, Sérgio akazu, Andre Rodrigues

**Affiliations:** 1 Department of Biochemistry and Microbiology, UNESP – São Paulo State University, Rio Claro, SP, Brazil São Paulo State University Rio Claro Brazil; 2 Center for the Studies of Social Insects, UNESP – São Paulo State University, Rio Claro, SP, Brazil São Paulo State University Rio Claro Brazil

**Keywords:** Hypocreales, Taxonomy, Phylogeny, Parasitic fungi, Symbiosis

## Abstract

*Escovopsis* (Ascomycota: Hypocreales, Hypocreaceae) is the only known parasite of the mutualistic fungi cultivated by fungus-growing ants (Formicidae: Myrmicinae: Attini: Attina, the “attines”). Despite its ecological role, the taxonomy and systematics of *Escovopsis* have been poorly addressed. Here, based on morphological and phylogenetic analyses with three molecular markers (internal transcribed spacer, large subunit ribosomal RNA and the translation elongation factor 1-alpha), we describe *Escovopsisclavatus* and *E.multiformis* as new species isolated from fungus gardens of *Apterostigma* ant species. Our analysis shows that *E.clavatus* and *E.multiformis* belong to the most derived *Escovopsis* clade, whose main character is the presence of conidiophores with vesicles. Nevertheless, the most outstanding feature of both new species is the presence of a swollen region in the central hypha of the conidiophore named swollen cell, which is absent in all previously described *Escovopsis* species. The less derived *Escovopsis* clades lack vesicles and their phylogenetic position within the Hypocreaceae still remains unclear. Considering the high genetic diversity in *Escovopsis*, the description of these new species adds barely two pieces to a huge taxonomic puzzle; however, this discovery is an important piece for building the systematics of this group of fungi.

## Introduction

Microorganisms play important roles in the stability of social insect colonies (Hughes et al. 2008, Joop and Vilcinskas 2016, Vanderpool et al. 2018). The environment of these insects has a high potential to harbour unique fungal species (Attili-Angelis et al. 2014, Harrington et al. 2014, Menezes et al. 2015, Montoya et al. 2016). The evolutionary success of the fungus garden of the fungus-farming ants (Formicidae: Myrmicinae: Attini: Attina, the “attines”) depends on complex symbiotic interactions amongst bacteria, fungi and the ants (Currie et al. 2003, Gerardo et al. 2006a, Kost et al. 2007). The association between attine ants and their mutualistic fungi (Basidiomycota: Agaricales) is the core of the attine colonies; however, *Escovopsis* (Ascomycota: Hypocreales: Hypocreaceae) can exploit this association. Although no specialised parasitic structures were found, studies showed that this parasite is able to kill the fungal cultivar as well as the ants and their mutualistic bacteria by chemical compounds (Currie 2001, Varanda-Haifig et al. 2017, Dhodary et al. 2018, Heine et al. 2018, Custodio and Rodrigues 2019). Despite the ecological relevance of *Escovopsis* as parasites of attine ant colonies, the taxonomy of this genus has been neglected.

Attine ants are classified in two sister clades: the Palaeoattina and Neoattina (Branstetter et al. 2017). Leafcutter ants (*Atta* and *Acromyrmex*) are considered the most derived attines within the Neoattina. Their behaviour is characterised by collecting fresh leaves and flowers to feed several cultivars from two clades of fungi in the Agaricaceae (Mueller et al. 2017, 2018). On the other hand, non-leafcutter ants also occur in both the Neoattina and Palaeoattina clades. Distinct from *Atta* and *Acromyrmex*, non-leafcutter ants collect seeds, insect frass and dry leaves to nourish a wide range of fungal cultivars in the Agaricaceae and Pterulaceae (Villesen et al. 2004, Schultz and Brady 2008).

The attine ant-fungus cultivar-*Escovopsis* symbiosis has been widely studied in leafcutter ants (Mueller and Gerardo 2002, Currie et al. 2003, Gerardo et al. 2004, 2006a, b). In addition to their contributions on the biology of *Escovopsis*, these studies also revealed considerable diversity of the parasite. Considering the variety of mutualistic fungi that non-leafcutter ants may cultivate, as well as the different substrates used for that purpose, a high diversity of *Escovopsis* species is unsurprising. This is especially true for *Apterostigma* (Gerardo et al. 2006b), a genus of non-leafcutter attine with species that cultivate different cultivars including *Leucoagaricusgongylophorus*, the domesticated fungus cultivated by many higher attine ant species, mostly leafcutter ants (Sosa-Calvo and Schultz 2010, Schultz et al. 2015, Ješovnik et al. 2016, Sosa-Calvo et al. 2017, Mueller et al. 2017, 2018).

While *Escovopsis* species exploiting gardens of *Atta*, *Acromyrmex*, *Trachymyrmex* and *Mycetophylax* were formally described, the morphological characters of the species associated with *Apterostigma* are unknown. A previous study associated clades of the parasite with the colour pattern of *Escovopsis* colonies (brown, yellow, white and pink; Gerardo et al. 2006b). However, no taxonomic studies were undertaken to formally describe these clades. Here, we describe *Escovopsisclavatus* and *E.multiformis* as new species isolated from the fungus garden of *Apterostigma*. The distinctive feature of these lineages is the presence of swollen cells at the base of the conidiophore branches. This phenotype differentiates these two new species from previously described *Escovopsis*. Considering that previous studies showed a high genetic diversity within *Escovopsis*, the description of these species adds two pieces to the enormous taxonomic puzzle which is *Escovopsis*.

## Material and methods

### Sampling sites and *Escovopsis* isolation

Five *Escovopsis* isolates were obtained from fungus gardens of five different colonies of *Apterostigma* spp. (Suppl. material 1: Table S1). The isolates LESF 847, LESF 853, LESF 854 and LESF 855 were obtained from colonies found in the Atlantic Rain Forest in Florianópolis, State of Santa Catarina, Brazil (October 2015). The isolate LESF 1136 was obtained from a colony found in the Amazon Forest in Cotriguaçu, State of Mato Grosso, Brazil (October 2017). The nests were found inside or under rotten logs. Fungus gardens, along with tending workers and brood, were collected in UV-sterilised plastic containers using sterilised spoon and forceps. Samples were taken to the Laboratory of Fungal Ecology and Systematics (LESF) at the UNESP – São Paulo State University, Rio Claro, Brazil.

For fungal isolation, seven garden fragments (0.5–1 mm^3^) were inoculated on plates (three plates per colony) containing potato dextrose agar (PDA, Neogen Culture Media, Neogen) supplemented with chloramphenicol (150 µg mL^-1^, Sigma) and incubated at 25 °C in darkness. Plates were monitored daily for fungal growth and, when *Escovopsis* mycelia sprouted, they were transferred to new PDA plates. All isolates were prepared as axenic (monosporic) cultures and stored under sterile distilled water kept at 8 °C (Castellani 1963) and at −80 °C (as conidia suspensions in 10% glycerol).

### Morphological analysis

The morphological characters of the five isolates (LESF 847, LESF 853, LESF 854, LESF 855 and LESF 1136) were examined. Due to the lack of standardisation of culture conditions for *Escovopsis*, the macroscopic characters of the colonies, i.e. radial growth, mycelium colour, morphology and presence of soluble pigments, were evaluated on eight different media: PDA, malt agar 2% [MA2%: 20 g L^-1^ of malt extract (Neogen Culture Media) and 15 g L^-1^ of agar (Neogen Culture Media)], cornmeal agar (CMD, Neogen Culture Media), synthetic nutrient agar [SNA: 1 g L^-1^ of KH_2_PO_4_ (Labsynth), 1 g L^-1^ of KNO_3_ (Labsynth), 0.5 g L^-1^ of MgSO_4_(7H_2_O) (Labsynth), 0.5 g L^-1^ of KCl (Labsynth), 0.2 g L^-1^ of Glucose (Labsynth), 0.2 g L^-1^ of Sucrose (Labsynth) and 15 g L^-1^ of Agar (Neogen Culture Media)], oatmeal agar (OA), potato carrot agar (PCA, HiMedia), malt extract agar 2% [MEA: 30 g L^-1^ of malt extract (Neogen Culture Media), 5 g L^-1^ of bacteriological peptone (Neogen Culture Media), 20 g L^-1^ of glucose (Labsynth) and 15 g L^-1^of Agar (Neogen Culture Media)] and Czapek yeast extract agar [CYA; 30 g L^-1^ of Sucrose (Labsynth), 5 g L^-1^ of Yeast extract (Neogen Culture Media), 1 g L^-1^ of KH_2_PO_4_ (Labsynth), 0.3 g L^-1^ of NaNO_3_ (Synth), 0.05 g L^-1^ of KCl (Labsynth), 0.05 g L^-1^ of MgSO_4_(7H_2_O) (Labsynth), 0.001 g L^-1^ of FeSO_4_ (Labsynth), 0.001 g L^-1^ of ZnSO_4_ (Labsynth), 0.0005 g L^-1^ of CuSO_4_ (Labsynth), 15 g L^-1^ of Agar (Neogen Culture Media)] at five temperatures (10 °C, 20 °C, 25 °C, 30 °C and 35 °C). These temperatures correspond to the conditions used in previous studies that described *Escovopsis* species (Seifert et al. 1995, Augustin et al. 2013, Masiulionis et al. 2015, Meirelles et al. 2015a). For this purpose, 200 μl of conidia were spread on plates with water-agar (WA) and incubated for seven days at 25 °C in darkness. Then, mycelium fragments of 0.5 cm diameter were cut from the WA plates and inoculated in the centre of the plates (90 × 15 mm) containing the eight culture media. All the strains examined showed better development in the dark and with unsealed Petri dishes to allow air passage; therefore, incubation was carried out in the darkness and without sealing the plates, for 14 days. Three replicate plates were inoculated for each media and for each incubation temperature.

To examine the microscopic characters, i.e. the morphology, size, branching patterns, vesicles and swollen cells of the conidiophores, as well as phialides and conidia, slide cultures on PDA and MEA were performed. Briefly, we placed a 5 mm^2^ fragment of culture medium on a microscopic slide and then we inoculated the fungus at the centre of the fragment. Then, the inoculated medium was covered with a coverslip and incubated at 25 °C for 4–7 days in the dark. After that, the coverslips, where the fungus grew, were removed and placed in new slides with a drop of lactophenol. Finally, the slides were examined under a light microscope (DM750, Leica, Germany). Fungal microscopic structures were photographed and measured (with 30 measurements per structure) in LAS EZ v.4.0 (Leica Application Suite).

Microscopic structures were also examined under scanning electron microscopy (SEM). Fungal samples (five days old cultures on PDA) were fixed in osmium tetroxide vapour for 72 h. Then, samples were dehydrated using a series of acetone concentrations (50, 75, 90, 95 and 100%) and dried to critical point using liquid CO_2_ (Balzers CPD030). The dried material was sputtered with gold (Balzers SCD050) and examined under the scanning electron microscope (TM3000, Hitachi).

### DNA extraction, PCR and sequencing

DNA extraction of the five strains was performed, following the steps published in Meirelles et al. (2015a). Three molecular markers were amplified: the internal transcribed spacer (ITS) region (White et al. 1990, Schoch et al. 2012); translation elongation factor 1-alpha (*tef*1) (Taerum et al. 2007); and the large subunit ribosomal RNA (LSU) (White et al. 1990, Haugland and Heckman 1998, Currie et al. 2003) (Suppl. material 1: Table S2).

PCR and sequence reaction conditions followed the steps published in Meirelles et al. (2015b) for the ITS region, Meirelles et al. (2015a) for *tef*1 and Augustin et al. (2013) for LSU. The final amplicons were cleaned up with Wizard SV Gel and PCR Clean-up System kit (Promega), following the manufacturer’s protocol. Sequences (forward and reverse) were generated in ABI3500 (Life Technologies). The LSU of 29 strains previously used in Meirelles et al. (2015b) was also amplified and sequenced for this study (Suppl. material 1: Table S1). The sequences were assembled in contigs in BioEdit v. 7.1.3 (Hall 1999) and deposited in GenBank (Suppl. material 1: Table S1 for accession numbers).

### Phylogenetic analyses

To infer the phylogenetic position of the new species in the *Escovopsis* clade, sequences from previous studies were retrieved from the GenBank and aligned with our new sequences in a dataset for each marker (Chaverri et al. 2003, Spatafora et al. 2007, Jaklitsch et al. 2011, Põldmaa 2011, Meirelles et al. 2015b). This data included sequences from the seven *Escovopsis* ex-type strains, from *Escovopsioidesnivea* and some species from *Hypomyces* and *Trichoderma*, as the phylogenetic closest relatives of *Escovopsis*. First, the three datasets [46 sequences of ITS (619 bp), LSU (594 bp) and *tef*1 (758 bp)] were aligned separately in MAFFT v.7 (Katoh and Standley 2013). The end parts of each alignment were removed manually by considering a point where the sequences presented greater homogeneity (all alignments are deposited in Treebase: http://purl.org/phylo/treebase/phylows/study/TB2:S23689). Then, a phylogenetic tree was inferred using each dataset separately. The nucleotide substitution model was selected by independent runs in jModelTest 2 (Darriba et al. 2012) using the Akaike Information Criterion with a 95% confidence interval. Second, the three datasets were concatenated using Winclada v.1.00.08 (Nixon 2002). The final file comprised 46 sequences totalling 1971 bp. All phylogenetic trees were reconstructed using maximum likelihood (ML) in RAxML v.8 (Stamatakis 2014) with 1000 independent trees and 1000 bootstrap replicates (MLB) and Bayesian Inference (BI) in MrBayes v.3.2.2. (Ronquist et al. 2012). The ML phylogenetic trees were reconstructed using the GTR + G substitution model and the BI phylogenetic trees were performed with the GTR + I + G substitution model. In the case of BI, two separate runs were carried out, each consisting of three hot chains and one cold chain and a Markov Chain Monte Carlo (MCMC) sampling for two million generations to obtain Bayesian posterior probability (PP) values for the clades. Convergence occurred when the standard deviation of split frequencies fell below 0.01 and the first 25% of the generations of MCMC sampling were discarded as burn-in. The final phylogenetic trees were edited in FigTree v.1.4 and in Adobe Illustrator CC v.17.1. *Lecanicilliumantillanum* CBS 350.85 was used as the outgroup in all trees, because it belongs to a family phylogenetically close to Hypocreaceae (Spatafora et al. 2007).

## Results

### Taxonomy

#### Escovopsis clavatus

Taxon classificationFungiHypocrealesHypocreaceae

Q.V. Montoya, M.J.S. Martiarena, D.A. Polezel, S. Kakazu & A. Rodrigues sp. nov.

MB828328

Figs 1 , 2 , 3

##### Etymology.

“*clavatus*” in reference to the predominantly clavate shape of vesicles.

##### Typification.

BRAZIL. Santa Catarina, Florianópolis, (27°44'39.6"S, 48°31'10.14"W), elev. 46 m, fungus garden, 08, 2015. *A. Rodrigues*. Holotype: CBS H-23845 (dried culture on PDA). Ex-type strain LESF 853 (= CBS 145326).

##### Sequences.

ITS (MH715096), *tef*1 (MH724270) and LSU (MH715110).

##### Description.

**Figure 1.**
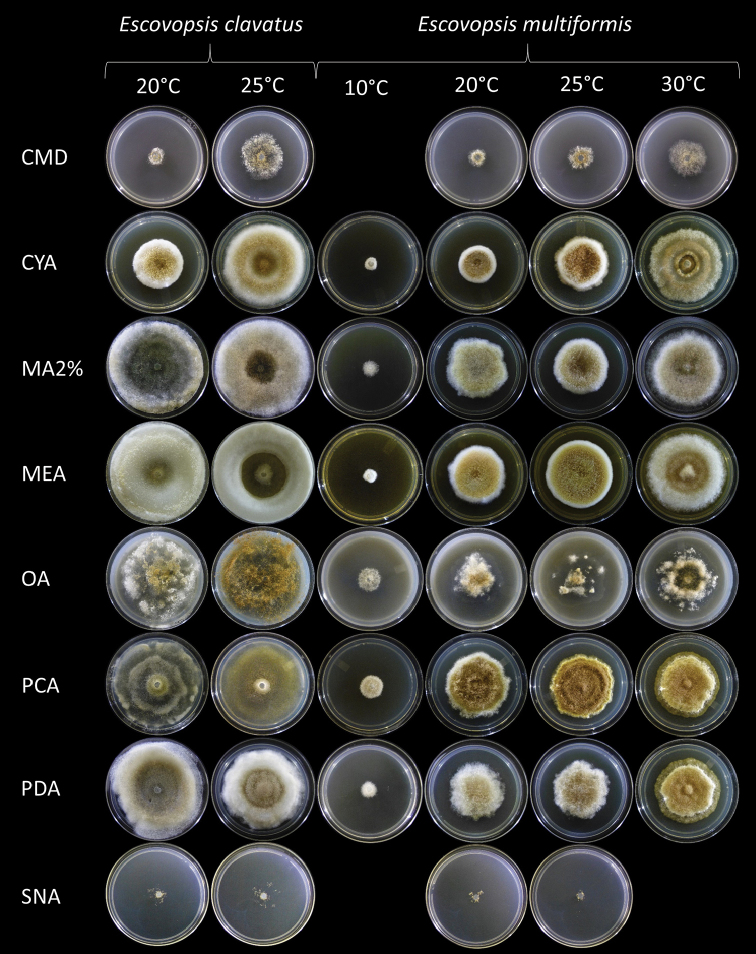
Colony macroscopic characters of *Escovopsisclavatus* and *Escovopsismultiformis* on CMD, CYA, MA2%, MEA, OA, PCA, PDA and SNA media after 14 days at 10, 20, 25 and 30 °C.

*Colonies* grow only at 20 and 25 °C (Fig. 1). At both temperatures, growth starts on the third day on CMD, CYA, MA2%, MEA, OA, PCA, PDA; and on the sixth day on SNA. Colonies have floccose aerial mycelia with a pale-brown colour after seven days. Faster growth was observed on MA2% and heavy sporulation was identified on MA2%, PDA and OA. At 20 °C, colonies reached 0.5–0.7 cm, 1.5–2.5 cm and 0.5–1 cm on CMD, CYA and SNA, respectively. At this temperature, colonies reached the edge of the plate after 10 days on MA2% and PCA; after 12 days on OA and MEA; and after 14 days on PDA and CYA. At 25 °C, colonies reach 2 cm, 3–3.2 cm and 2 cm on CMD, CYA and SNA, respectively, after 14 days. At this temperature, colonies reached the plate edge after seven days on OA and PCA; and after 10 days on MA2%, MEA and PDA. Concentric rings were observed only on PCA at 20 °C (Fig. 1). No pustule-like structures were observed.

**Figure 2.**
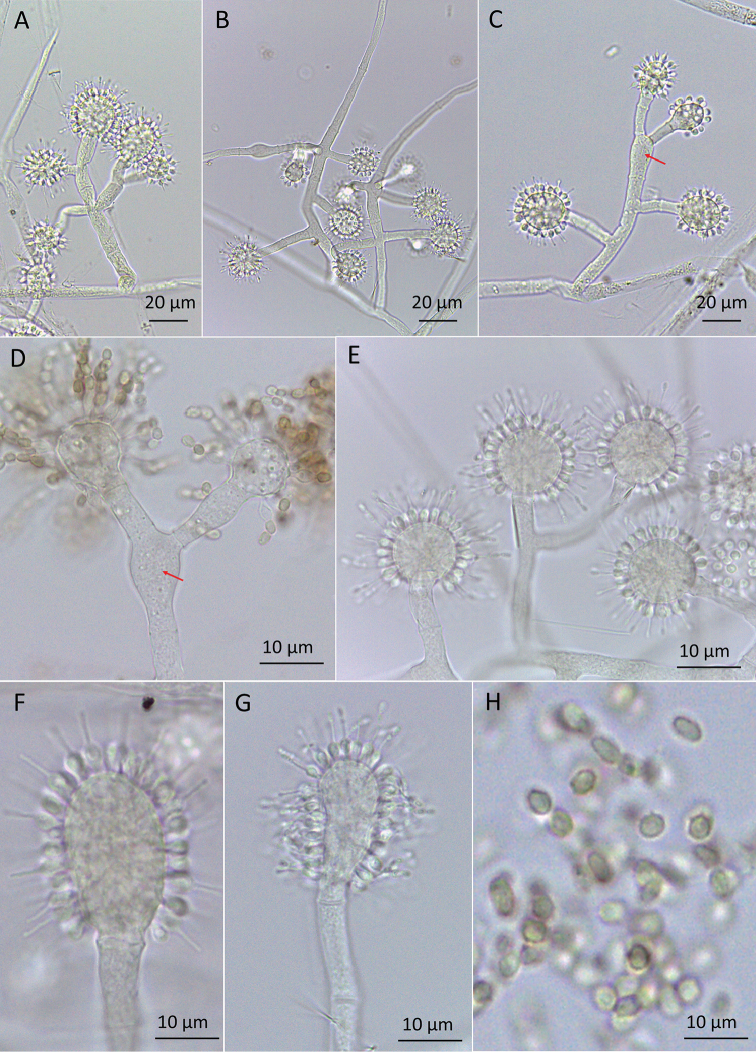
*Escovopsisclavatus*. **A, B** Conidiophores without “swollen cells” **C, D** Conidiophores with “swollen cells” (red arrows) **E–G** Vesicles in various shapes with phialides pattern **G** Conidia.

**Figure 3.**
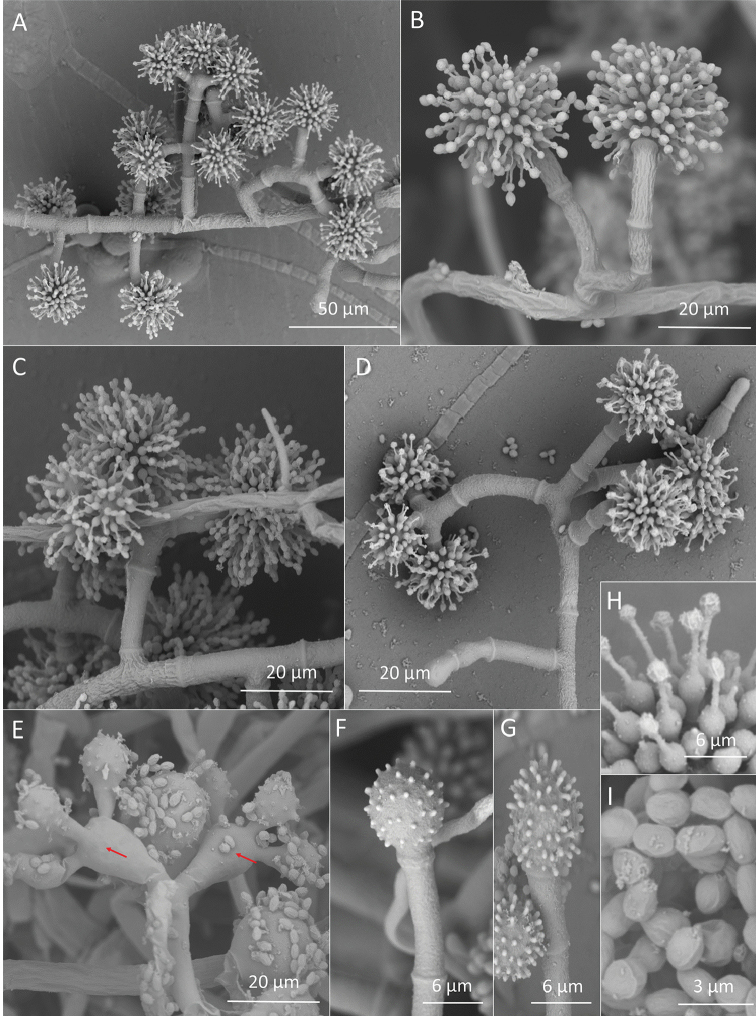
*Escovopsisclavatus*. SEM images **A–D** Conidiophores without “swollen cells” **E** Conidiophore with “swollen cells” (red arrows) **F, G** Vesicles **H** Phialides **G** Conidia.

*Conidiophores* arising from aerial hypha alternated or opposite (Fig. 2A), with the main axis of 50–780 μm in length, some without branching and often with 1–2 levels of branching (Figs 2A, E, 3A, E). Branches arise from the main axis of the conidiophore in an alternated or opposite pattern, with a septum near to the central axis and before the vesicle, usually with 1–2 branches at each branching point (16–138 μm long) or 2–4 branches arising from swollen cells (28–35 μm long), mostly forming angles less than 90° and less frequently right angles, usually straight and sometimes slightly curved up or down. Each branch terminates in a vesicle, with 1–8 fertile heads per conidiophore. Swollen cells are present in 15% of the total of conidiophores examined (Figs 2C, D, 3E) and can measure 10–18 μm long × 7–9 μm wide. *Vesicles* with only a septum at the base, in various shapes: globose (8%), subglobose (24%), broadly ellipsoidal / clavate (33%), ellipsoidal (27%), cylindrical (8%) (Figs 2E–G and 3F–G); and reaching 9–27 μm long × 7–20 μm wide. *Phialides* lageniform formed on vesicles (Fig. 3H), with 5–8 μm in total length, elongated base (0.5–1.5 μm × 0.5–1 μm), followed by a swollen section (1.5–2.5 μm × 1–3 μm) and a thin neck (1.5–4 μm × 0.5 μm). *Conidia* with 1.5 μm–2.5 μm long × 0.5 μm–1.5 μm wide, in various shapes: broadly ellipsoidal (5%), ellipsoidal (43.3%), cylindrical (51.7%); brown, with smooth and slightly thickened walls and in chains (Figs 2H, 3I).

##### Habitat.

Isolated from fungus gardens of *Apterostigma* sp.

##### Additional specimens examined.

BRAZIL. Santa Catarina, Florianópolis, (27°44'38.94"S, 48°31'9.3"W), elev. 32 m, fungus garden, 08, 2015. *A. Rodrigues*. LESF 854 (ITS – MH715097, *tef*1 – MH724271 and LSU – MH715111). Santa Catarina, Florianópolis, (27°44'39.49"S, 48°31'9.72"W), elev. 38 m, fungus garden, 08, 2015. *A. Rodrigues*. LESF 855 (ITS – MH71509, *tef*1 – MH724272 and LSU – MH715112).

##### Notes.

*Escovopsisclavatus* is phylogenetically closely related to *E.multiformis* and its most distinctive characters are its growth temperatures, the conidiophore branching and the swollen cells. It grows at 20 and 25 °C; nevertheless, *E.multiformis* grows at 10, 20, 25 and 30 °C. The conidiophore of *E.clavatus* is larger and more branched than the conidiophore of *E.multiformis*. In addition, the swollen cells of *E.clavatus* are less frequent and shorter than in *E.multiformis*. The character distinguishing *E.clavatus* from other species of *Escovopsis* is the swollen cell on the conidiophores and because it is phylogenetically placed in a distinct clade.

#### Escovopsis multiformis

Taxon classificationFungiHypocrealesHypocreaceae

Q.V. Montoya, M.J.S. Martiarena, D.A. Polezel, S. Kakazu & A. Rodrigues sp. nov.

MB828329

Figs 1 , 4 , 5

##### Etymology.

“*multiformis*” in relation to the different vesicle shapes found in the same isolate.

##### Typification.

BRAZIL. Santa Catarina, Florianópolis, (27°28'11.28"S, 48°22'39.48"W), elev. 119 m, fungus garden, 08, 2015. *A. Rodrigues*. Holotype: CBS H-23846 (dried culture on PDA). Ex-type strain LESF 847 (= CBS 145327).

##### Sequences.

ITS (MH715091), *tef*1 (MH724265) and LSU (MH715105).

##### Description.

*Colonies* grow at 10, 20, 25 and 30 °C (Fig. 1). The best growth temperature was 30 °C. At this temperature, colonies reached 1.2–1.4 cm, 2.7–3 cm, 2.6–3 cm, 3.3–3.5 cm, 2.5–2.8 cm, 2.7–2.9 cm and 1.9–2.5 cm in radius on CMD, CYA, MA2%, MEA, OA, PCA and PDA, after 14 days, respectively. Colonies exhibit light-brown floccose mycelia (colony edge usually lighter or white). The colour shades and the character of the aerial mycelium vary on each culture medium (Fig. 1). Colonies present concentric rings with a hardened ring similar to a crust in the centre on CYA (Fig. 1) and the sporulation is more abundant on PCA and PDA. At 20 °C, on CMD, CYA, MA2%, MEA, OA, PCA, PDA and SNA, colonies attained 0.5–0.8 cm, 1.1–2.2 cm, 2–2.5 cm, 2.1–2.3 cm, 2–2.5 cm, 2.8 cm, 1.9–2.4 cm and 0–0.1 cm in radius, respectively. At 25 °C, colonies reached 1 cm, 2.1–2.3 cm, 2–2.4 cm, 2.5–2.6 cm, 2.2–2.7 cm, 2.8–3 cm, 1.8–2 cm and 0.1–0.2 cm in radius on CMD, CYA, MA2%, MEA, OA, PCA, PDA and SNA, respectively. Pustule-like structures were observed on OA and CMD at 20, 25 and 30 °C. At 10 °C, the colony growth was inconspicuous, reaching 0.2–0.3 cm, 0.2–0.4 cm, 0.3 cm, 0.6–0.8 cm, 0.8 cm and 0.3–0.5 cm in radius on CYA, MA2%, MEA, OA, PCA and PDA, respectively, after 14 days. At this temperature, growth started in these culture media after seven days and sporulation occurred only after the 12^th^ day. No growth was observed at 35 °C.

**Figure 4.**
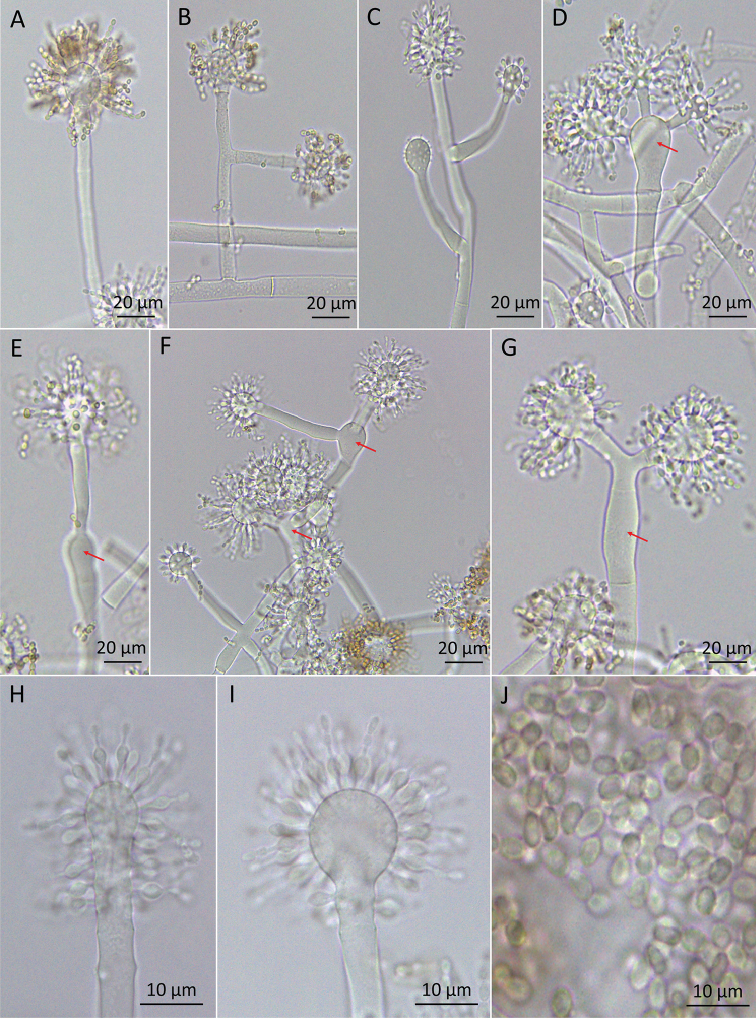
*Escovopsismultiformis*. **A–C** Conidiophores mono- and polycephalous without “swollen cells” **D–G** Conidiophores mono and polycephalous with “swollen cells” (red arrows) **H, I** Vesicles in various shapes **J** Conidia.

**Figure 5.**
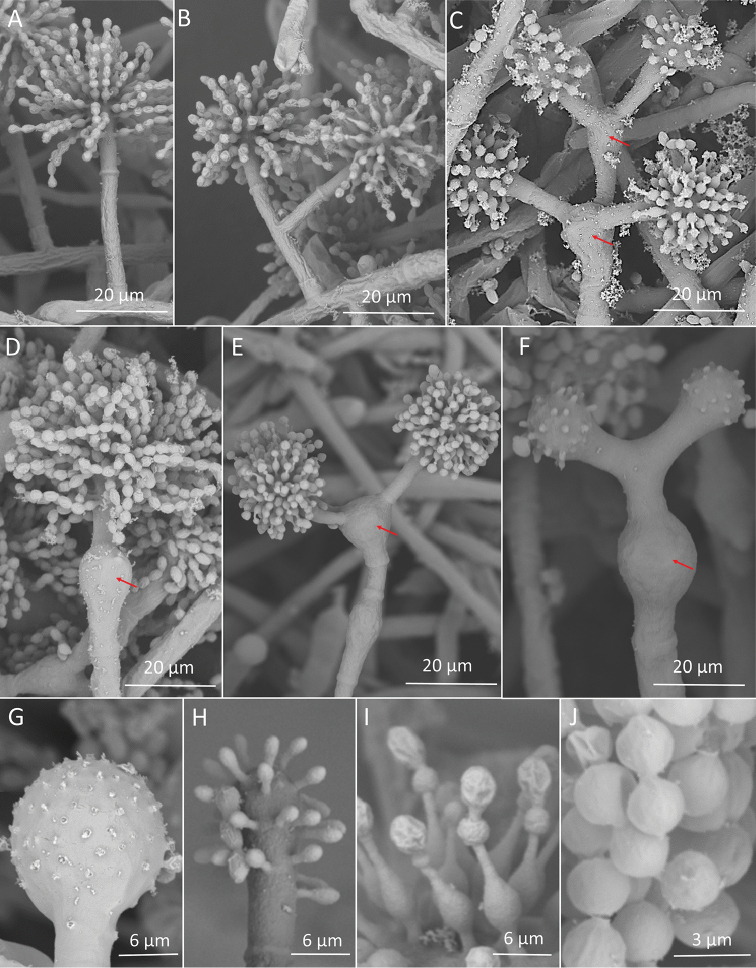
*Escovopsismultiformis*. SEM images **A, B** Conidiophores mono- and polycephalous without “swollen cells” **C–F** Conidiophores mono- and polycephalous with “swollen cells” (red arrows) **G, H** Vesicles **I** Phialides **J** Conidia.

*Conidiophores* arising from aerial hypha alternated or opposite (Fig. 3A), with the main axis of 41–293 μm in length, some without branching and most of them with one level of branching. Rarely, branches form two levels branching (Figs 4A–C, 5A, B). Branches arise from the main axis of the conidiophore alternated, with a septum near the central axis and before the vesicle, usually with one branch at each branching point (32–84 μm long) or 2–4 branches arising from swollen cells (17–86 μm long), mostly forming right angles, usually slightly curved up. Each branch terminates in a vesicle, with 1–4 fertile heads per conidiophore. Swollen cells are present in 27% of the total of conidiophores examined (Figs 4D–G, 5C–F) and can measure 16–34 μm long × 9–20 μm wide. Sometimes, one swollen cells’ branch gives rise to another swollen cell with more branches (Figs 2F, 3C). *Vesicles* with only a septum at the base, in various shapes: globose (22%), subglobose (37%), broadly ellipsoidal (26%), ellipsoidal (10%), cylindrical (5%) (Figs 4H, I, 5G, H); and reaching 12–27 μm × 9–17 μm wide. *Phialides* lageniform formed on vesicles (Fig. 5I), with 6–10 μm in total length, elongated base (1– 2.5 μm × 0.5–1μm), followed by a swollen section (2.5–4.5 μm × 2–3.5 μm) and a thin neck (1– 4.5 μm × 0.5–1 μm). *Conidia* are 2.5–3.5 μm long × 1.5–2.5 μm wide, in various shapes: globose (2%), subglobose (3%), broadly ellipsoidal (33%), ellipsoidal (47%), cylindrical (15%); brown, with smooth and slightly thickened walls and in chains (Figs 4, 5J).

##### Habitat.

Isolated from fungus garden of *Apterostigma* sp.

##### Additional specimens examined.

BRAZIL. Mato Grosso, Cotriguaçu, (09°49'22.74"S, 58°15'32.04"W), elev. 252 m, fungus garden, 10, 2017. *Q. V. Montoya*. LESF 1136 (ITS – MH715092, *tef*1 – MH724266 and LSU – MH715106).

##### Notes.

*Escovopsismultiformis* is closely related to *E.clavatus*. Different from *E.clavatus* that grow at 20 and 25 °C, *E.multiformis* grow at 10, 20, 25 and 30 °C. The optimum growth temperature of *E.multiformis* is 30 °C and that of *E.clavatus* is 25 °C. The conidiophores of *E.multiformis* are smaller and less branched than *E.clavatus* and the swollen cells are more frequent and larger than those found in *E.clavatus*. *E.multiformis* differs from other described species by the presence of conidiophores with a swollen cell, the presence of different vesicles shapes and because it is phylogenetically placed in a distinct clade.

### Morphological analyses

The isolates LESF 853 (*Escovopsisclavatus*, Figs 1–3) and LESF 847 (*Escovopsismultiformis*, Figs 1, 4, 5) differed from the seven previously described *Escovopsis* species, mainly in micro-morphological structures. All isolates had white colonies with a floccose appearance on all culture media, but *E.clavatus* had the most floccose colonies. After 5–7 days incubation, the centre of the colonies turned pale brown and, after 7 days, the entire colony gradually turned from white to light brown (not always from the middle to the edge in *E.multiformis*).

*Escovopsismultiformis* showed growth at wide ranges of temperature (from 10–30 °C); nonetheless, *E.clavatus* showed growth only at 20 and 25 °C (Fig. 1). None of the isolates grew at 35 °C. On all culture media, the best growth was obtained at 25 °C for *E.clavatus* and at 30 °C for *E.multiformis*. In all cases where growth was observed, it started between 24 to 36 hours and sporulation started on the third day.

All strains of both species have a unique type of conidiophore with a swollen cell, from which branches emerge (Figs 2C, D, 3E, 4D–F, 5C–F). These conidiophores were more frequent in *E.multiformis* than *E.clavatus* (27% and 15%, respectively). Mono or polycephalous conidiophores, without the swollen cells, that were described in the other *Escovopsis* species, were also present but with some differences in the size and branching pattern (Figs 2A, B, 3A–D, 4A–C, 5A, B). Conidiophores with cruciform or opposed branches were rarely observed. On the other hand, the two new species had basipetal and smooth-walled conidia with slightly thickened walls, formed from phialides. No chlamydospores were observed in the aerial or submersed mycelia of any of the strains.

### Phylogenetic analyses

**Figure 6.**
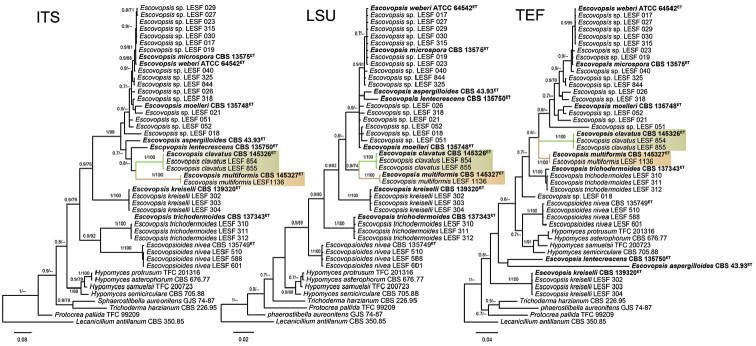
Phylogenetic position of *Escovopsisclavatus* and *Escovopsismultiformis* considering each molecular marker separately (ITS, LSU and *tef1*). The trees were reconstructed under Bayesian and Maximum Likelihood inferences. The numbers on branches indicate the posterior probabilities and the bootstrap support values, respectively. The seven *Escovopsis* ex-type strains are denoted in bold and the new species are highlighted in green (*E.clavatus*) and light brown (*E.multiformis*). The trees include a total of 46 *Escovopsis* sequences of each marker (ITS – 619 bp, LSU – 594 bp and *tef*1 – 758 bp) and *Escovopsioides*, *Hypomyces*, *Sphaerostilbella*, *Trichoderma* and *Protocrea* were included as the closest phylogenetic relatives of *Escovopsis*. *Lecanicilliumantillanum* CBS 350.85 was used as the outgroup. ET: ex-type.

Separate phylogenetic analyses with the three molecular markers showed topological differences because of the incongruity placement of the formal described *Escovopsis* species and some strains that form new phylogenetic clades within the genus (Fig. 6). The phylogenetic placement of *E.multiformis* and *E.clavatus* also presented conflicts amongst the three molecular markers; however, the position of each strain that made up both new species was concordant through the three genealogies (PP= 1; MBL= 100%, Fig. 6).

**Figure 7.**
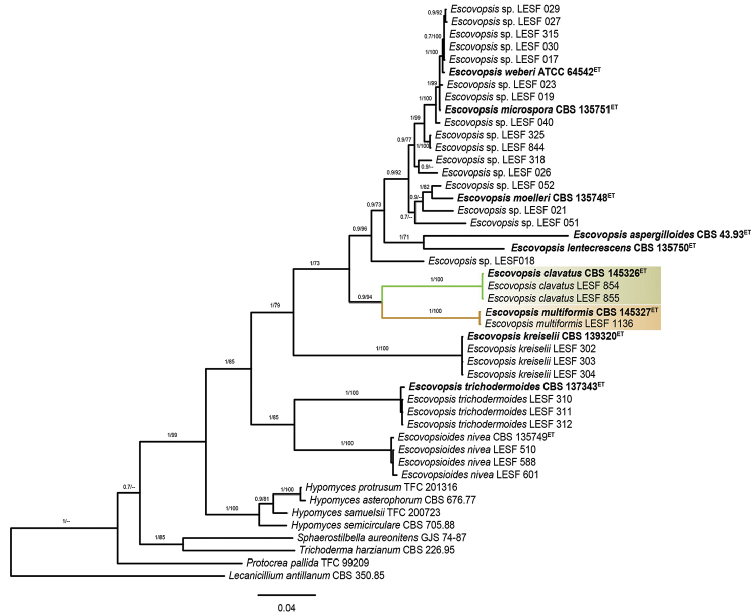
Phylogenetic position of *Escovopsisclavatus* and *Escovopsismultiformis*. The phylogenetic analysis is based on the concatenated sequences of ITS, LSU and *tef1*; and the tree was reconstructed using Bayesian and Maximum Likelihood inferences. Numbers on branches indicate the posterior probabilities and the bootstrap support values, respectively. All *Escovopsis* species previously described are denoted in bold and the new species are highlighted in green (*E.clavatus*) and light brown (*E.multiformis*). The tree includes a total of 40 *Escovopsis* sequences with 1971 bp (ITS – 619 bp, LSU – 594 bp and *tef*1 – 758 bp). The data also included sequences from *Escovopsioides*, *Hypomyces*, *Sphaerostilbella*, *Trichoderma* and *Protocrea* as the closest phylogenetic relatives of the parasite. *Lecanicilliumantillanum* CBS 350.85 was used as the outgroup. ET: ex-type strains. Bar: 0.04 substitutions per nucleotide position.

The combined analysis also confirmed *E.multiformis* and *E.clavatus* as two new phylogenetic species in *Escovopsis* (PP= 1; MLB= 100%, Fig. 7) and showed the strain LESF 018 (a vesiculated *Escovopsis* species) as the closest relative of both. Nevertheless, the concatenated BI and ML trees also presented few differences between them with respect to the position of the *E.aspergilloides* and *E.lentecrescens*. The BI analysis placed *E.aspergilloides* and *E.lentecrescens* separate from *E.multiformis* and *E.clavatus* (Fig. 7); however, the ML analysis showed the former species as sister clades of *E.multiformis* and *E.clavatus*.

It is important to highlight that the concatenated analysis, as well as the trees inferred with ITS and LSU, showed the vesiculated *Escovopsis* (*E.aspergilloides*, *E.clavatus*, *E.lentecrescens*, *E.microspora*, *E.moelleri*, *E.multiformis*, *E.weberi*) as the most derived group, separated from the non-vesiculated *Escovopsis* (*E.kreiselii* and *E.trichodermoides*). In addition, both the combined and the analysis performed with ITS and *tef*1 showed some *Escovopsis* species (*E.aspergilloides*, *E.kreiselii*, *E.lentecrescens* and *E.trichodermoides*) often clustering with other Hypocreaceae genera or falling outside the *Escovopsis* clade, which reveals that *Escovopsis* is apparently paraphyletic (Figs 6, 7).

## Discussion

The attine ants have persisted for millions of years because of the biological relationships that these insects maintain with the beneficial microorganisms that inhabit their colonies. Several studies tried to understand how these biological relationships sculptured the evolutionary history of the attines (Mueller et al. 1998, Currie et al. 2003, Gerardo et al. 2006ab, Nygaard et al. 2016, Mueller et al. 2018). Nevertheless, the taxonomy of *Escovopsis*, the only known parasite in the attine’s colony, has been poorly addressed. Considering that *Escovopsis* co-evolved with the attine ants’ cultivar, improved knowledge about the taxonomy and systematics of this genus could shed light on the evolutionary success of these insects. Therefore, the discovery and description of new *Escovopsis* species is an important advance in understanding this system.

Subsequent to the formal description of *Escovopsis* (Muchovej and Della Lucia 1990), several studies showed a high genetic diversity of this genus in the colonies of both leafcutter and non-leafcutter attine ants (Gerardo et al. 2006a, Meirelles et al. 2015b). However, only seven species of the parasite have been described so far (Seifert et al. 1995, Augustin et al. 2013, Masiulionis et al. 2015, Meirelles et al. 2015a) and the morphological diversity and physiology of the parasite remain unknown. In addition, a lack of standardised conditions for describing the morphology of *Escovopsis* hinders researchers from identifying morphological characters that might help to distinguish *Escovopsis* species from one another and from the other related genera from the Hypocreaceae. Unfortunately, this fact made it difficult to describe new species of the parasite. Studies showed that the expressed phenotypic characters (phenotypic plasticity) of fungi are directly influenced by growth conditions (Slepecky and Starmer 2009, Sharma and Pandey 2010, Wrzosek et al. 2017, Kim et al. 2017). As the morphological plasticity of *Escovopsis* species is still poorly understood, the standardisation of cultivation conditions is imperative. The strains described as new species here were evaluated on eight different culture media (those used in the description of the seven previous species) and at five temperatures (to establish cardinal growth temperatures). Due to the lack of standard culture conditions, the comparison with each species previously described was only partial. Nonetheless, we are providing characters of these two new species in all the conditions previously used, to help future researchers to standardise the taxonomy of the genus.

Recent attempts to expand the morphological concept of *Escovopsis* generated inconsistencies in the taxonomy and systematics of this genus (Masiulionis et al. 2015, Meirelles et al. 2015a). The morphological characters that initially gave rise to the concept of *Escovopsis* (presence of terminal vesicles and phialidic conidiogenesis, see Muchovej and Della Lucia 1990) are distinctive to delineate *Escovopsis*, because no other genus in the Hypocreaceae family has such combined characters. However, some *Escovopsis* species described recently, namely *E.trichodermoides* and *E.kreiselii*, lack vesicles and each has a different kind of conidiogenesis (synchronous and sympodial, respectively). Besides, the results of the phylogenetic analysis performed in previous studies (Meirelles et al. 2015b, Masiulionis et al. 2015, Augustin et al. 2013), as well as the results from our analysis, reveal that *Escovopsis* is paraphyletic (Figs 6, 7). Therefore, future studies will have to reconsider if both species indeed belong to *Escovopsis*. For this purpose, the taxonomic conditions need to be delimited and additional molecular markers will have to be included to help resolve those phylogenetic incongruities. Then the generic concept of *Escovopsis* species should be revisited.

Our study shows that the ex-type strains LESF 853 (*E.clavatus*) and LESF 847 (*E.multiformis*) form a monophyletic clade within most derived *Escovopsis* (vesiculated *Escovopsis*, PP = 1, BML = 100%). Most interesting, unlike the other *Escovopsis* species, the two new species present a unique type of conidiophore with a swollen cell, from which one to four branches arise. The newly described species also possess smooth conidia with slightly thickened walls. A recent study suggests the possibility that conidia ornamentation could be associated with the mechanism for horizontal transmission of *Escovopsis* between ant colonies and with the latency of the parasite conidia. This hypothesis was based on observations of some conidia adhering to the ant legs and in spore dormancy in vitro bioassays (Augustin et al. 2017). The same authors also argued that such character could be used as morphological markers for the taxonomy of the genus. However, because of scarce knowledge of the morphological features of the *Escovopsis* species, it is difficult to decipher which phenotypic character could be considered diagnostic for this genus. Therefore, future researchers need to carefully evaluate the phenotypic characters of each *Escovopsis* clade to determine which of characters are homologous versus those that are homoplasious to build taxonomic keys.

Considering the high genetic diversity of *Escovopsis* and the poor knowledge of its taxonomy, our study suggests that the fungus gardens of attine ants host a great diversity of *Escovopsis* that has yet to be discovered. Thus, the description of these new species are merely two small pieces of a complex puzzle. Nonetheless, our work should help future researchers to build the framework for the systematics of this parasitic fungus.

## Supplementary Material

XML Treatment for Escovopsis clavatus

XML Treatment for Escovopsis multiformis

## Supplementary materials

Supplementary material 1 Supplementary tables Data type: phylogenetic data Explanation note: Table S1. *Escovopsis* strains used in the phylogenetic analyses and their associated metadata. Table S2. Molecular markers, primers and PCR conditions used in this study. This is an open access article distributed under the terms of the Creative Commons Attribution License (CC BY 4.0), which permits unrestricted use, distribution, and reproduction in any medium, provided the original author and source are credited.Quimi Vidaurre Montoya, Maria Jesus Sutta Martiarena, Danilo Augusto Polezel, Sérgio Kakazu, Andre Rodrigues
