# Supplementary material for: More pieces to a huge puzzle: Two new Escovopsis species from fungus gardens of attine ants
Source: MycoKeys. 2019 Feb 18;(46):97–118. doi: 10.3897/mycokeys.46.30951 (PMC6389644; doi:10.3897/mycokeys.46.30951)
Supplement: Supplementary material 1 [file mycokeys-46-097-s001.docx]

**Supplementary material**

**More pieces to the huge puzzle: two new *Escovopsis* species from fungus gardens of attine ants**

Quimi Vidaurre Montoya, Maria Jesus Sutta Martiarena, Danilo Augusto Polezel, Sérgio Kakazu, Andre Rodrigues

Table S1. *Escovopsis* strains used in the phylogenetic analyses and their associated metadata.

| **Fungal species** | **Strain ID** | **Specimen voucher** | **City, State, Country** | **GPS** | **Habitat** | **Ant colony ID** | **GenBank accession number**s | | | **Reference** |
| --- | --- | --- | --- | --- | --- | --- | --- | --- | --- | --- |
|  |  |  |  |  |  |  | **ITS** | **LSU** | ***tef*1** |  |
| *Escovopsis microspora* | CBS 135751^ET^ | VIC:31756 | Viçosa, Minas Gerais, Brazil | 20°44'31.71''S 42°52'43.83''W | Fungus garden of *Acromyrmex subterraneus molestans* | --- | JQ815076 | KF293284 | KJ935030 | Augustin et al. (2013) |
| *Escovopsis weberii* | ATCC 64542 ^ET^ | --- | Viçosa, Minas Gerais, Brazil | --- | Carpenter ant fungal mass | --- | KF293285 | KF293281 | KF293275 | Augustin et al. (2013) |
| *Escovopsis moelleri* | CBS 135748 ^ET^ | VIC:31753 | Viçosa, Minas Gerais, Brazil | 20°44'31.71''S 42°52'43.83''W | Fungus garden of *Acromyrmex subterraneus molestans* | --- | JQ815077 | JQ855715 | JQ855712 | Augustin et al. (2013) |
| *Escovopsis* *aspergilloides* | CBS 423.93 ^ET^ | DAOM:216382 | Trinidad and Tobago: Trinidad | --- | Nest of *Trachymyrmex ruthae* | --- | NR_137160 | KF293283 | KF293277 | Seifert et al. (1995) |
| *Escovopsis lentecrescens* | CBS 135750 ^ET^ | VIC:31755 | Viçosa, Minas Gerais, Brazil | 20°44'31.71''S 42°52'43.83''W | Fungus garden of *Acromyrmex subterraneus subterraneus* | --- | JQ815079 | JQ855717 | JQ855714 | Augustin et al. (2013) |
| *Escovopsis multiformis* | CBS 145327 ^ET^ | 1606w | Florianópolis, Santa Catarina, Brazil | 27°28'11.28''S 48°22'39.48''W | Fungus garden of *Apterostigma* sp. | AR150816-06 | MH715091 | MH715105 | MH724265 | This study |
| *Escovopsis multiformis* | LESF 1136 | QVM277 | Alta Floresta, Mato Grosso, Brazil | 09°49'22.74''S 58°15'32.04''W | Fungus garden of *Apterostigma* sp. | QVM171004-02 | MH715092 | MH715106 | MH724266 | This study |
| *Escovopsis clavatus* | CBS 145326 ^ET^ | 1707 | Florianópolis, Santa Catarina, Brazil | 27°44'39.6''S 48°31'10.14''W | Fungus garden of *Apterostigma* sp. | AR150817-07 | MH715096 | MH715110 | MH724270 | This study |
| *Escovopsis clavatus* | LESF 854 | 1704A | Florianópolis, Santa Catarina, Brazil | 27°44'38.94''S 48°31'9.3''W | Fungus garden of *Apterostigma* sp. | AR150817-04A | MH715097 | MH715111 | MH724271 | This study |
| *Escovopsis clavatus* | LESF 855 | 1705B | Florianópolis, Santa Catarina, Brazil | 27°44'39.49''S 48°31'9.72''W | Fungus garden of *Apterostigma* sp. | AR150817-05B | MH715098 | MH715112 | MH724272 | This study |
| *Escovopsis kreiselii* | CBS 139320 ^ET^ | LESF 053 | Florianópolis, Santa Catarina, Brazil | 27°37'50.01''S 48°27'03.64''W | Fungus garden of *Mycetophylax morchi* | AR090306–01 | KJ808767 | HJ808765 | KJ 808766 | Meirelles et al. (2015a) |
| *Escovopsis kreiselii* | LESF 302 | AR14022705 | Florianópolis, Santa Catarina, Brazil | 27°31'24.96''S  48°25'3.78''W | Fungus garden of *Mycetophylax morchi* | AR140227–05 | MH715085 | MH715099 | MH724259 | This study |
| *Escovopsis kreiselii* | LESF 303 | AR14022705B | Florianópolis, Santa Catarina, Brazil | 27°31'24.96''S  48°25'3.78''W | Fungus garden of *Mycetophylax morchi* | AR140227–05 | MH715086 | MH715100 | MH724260 | This study |
| *Escovopsis kreiselii* | LESF 304 | AR14022705T2D | Florianópolis, Santa Catarina, Brazil | 27°31'24.96''S  48°25'3.78''W | Fungus garden of *Mycetophylax morchi* | AR140227–05 | MH715087 | MH715101 | MH724261 | This study |
| *Escovopsis trichodermoides* | CBS 137343 ^ET^ | VEM-2014 | Florianópolis, Santa Catarina, Brazil | 22°23'46.93''S, 47°32'40.12''W | Fungus garden of *Mycocepurus goeldii* | --- | KJ485699 | MF116052 | KF033128 | Masiulionis et al. (2015) |
| *Escovopsis trichodermoides* | LESF 310 | AR14022604A1 | Florianópolis, Santa Catarina, Brazil | 27°37'49.62''S, 48°27'3.6''W | Fungus garden of *Mycetophylax morchi* | AR140226-04A | MH715088 | MH715102 | MH724262 | This study |
| *Escovopsis trichodermoides* | LESF 311 | AR14022604A2 | Florianópolis, Santa Catarina, Brazil | 27°37'49.62''S, 48°27'3.6''W | Fungus garden of *Mycetophylax morchi* | AR140226-04A | MH715089 | MH715103 | MH724263 | This study |
| *Escovopsis trichodermoides* | LESF 312 | AR14022604ALA | Florianópolis, Santa Catarina, Brazil | 27°37'49.62''S, 48°27'3.6''W | Fungus garden of *Mycetophylax morchi* | AR140226-04A | MH715090 | MH715104 | MH724264 | This study |
| *Escovopsis* sp. | LESF 017 | NL001 | Botucatu, São Paulo, Brazil | 22° 50.774' S 48° 26.160'W | Midden of *Atta capiguara* | N66 | KM817072 | MH715113 | KM817142 | Meirelles et al. (2015b) |
| *Escovopsis* sp. | LESF 018 | NL002 | Botucatu, São Paulo, Brazil | 22° 50.774' S 48° 26.160'W | Fungus garden of *Atta capiguara* | N66 | KM817073 | MH715114 | KM817143 | Meirelles et al. (2015b) |
| *Escovopsis* sp. | LESF 019 | NL005 | Botucatu, São Paulo, Brazil | --- | Fungus garden of *Atta sexdens rubropilosa* | N68 | KM817074 | MH715115 | KM817144 | Meirelles et al. (2015b) |
| *Escovopsis* sp. | LESF 021 | ES002 | Rio Claro, São Paulo, Brazil | --- | Fungus garden of *Atta sexdens rubropilosa* | N-Ale | KM817053 | MH715116 | KM817123 | Meirelles et al. (2015b) |
| *Escovopsis* sp. | LESF 023 | ES005 | Alta Floresta, Mato Grosso, Brazil | --- | Fungus garden of *Atta cephalotes* | SES040129-06 | KM817056 | MH715117 | KM817126 | Meirelles et al. (2015b) |
| *Escovopsis* sp. | LESF 026 | ES009 | Carreiro da Várzea, Amazonas, Brazil | --- | Fungus garden of *Atta cephalotes* | SES040220-01 | KM817060 | MH715118 | KM817130 | Meirelles et al. (2015b) |
| *Escovopsis* sp. | LESF 027 | ES010 | Rio Claro, São Paulo, Brazil | --- | Fungus garden of *Acromyrmex landolti* | --- | KM817061 | MH715119 | KM817131 | Meirelles et al. (2015b) |
| *Escovopsis* sp. | LESF 029 | ES012 | Corumbataí, São Paulo, Brazil | --- | Fungus garden of *Atta sexdens* | 13B | KM817063 | MH715120 | KM817133 | Meirelles et al. (2015b) |
| *Escovopsis* sp. | LESF 030 | ES013 | Corumbataí, São Paulo, Brazil | --- | Fungus garden of *Atta sexdens* | 1 | KM817064 | MH715121 | KM817134 | Meirelles et al. (2015b) |
| *Escovopsis* sp. | LESF 040 | RS020 | Nova Petrópolis, Rio Grande do Sul, Brazil | --- | Fungus garden of *Acromyrmex laticeps* | AOMB060904-05 | KM817077 | MH715122 | EU082803 | Meirelles et al. (2015b) |
| *Escovopsis* sp. | LESF 051 | SES009 | Palmeiras, Bahia, Brazil | --- | Fungus garden of *Trachymyrmex* sp. | SES081108-04 | KM817092 | MH715123 | KM817153 | Meirelles et al. (2015b) |
| *Escovopsis* sp. | LESF 052 | SES010 | Camp 41, Manaus, Amazonas, Brazil | --- | Fungus garden of *Trachymyrmex diversus* | SES090109-04 | KM817093 | MH715124 | KM817154 | Meirelles et al. (2015b) |
| *Escovopsis* sp. | LESF 315 | NL007 | Botucatu, São Paulo, Brazil | --- | Fungus garden of *Atta sexdens rubropilosa* | N68 | KM817075 | MH715125 | KF240730 | Meirelles et al. (2015b) |
| *Escovopsis* sp. | LESF 318 | ES029 | Palmas, Tocantins, Brazil | --- | Fungus garden of *Trachymyrmex* sp. | WGPM091021-01 | KM817069 | MH715126 | KM817139 | Meirelles et al. (2015b) |
| *Escovopsis* sp. | LESF 325 | BA004 | Camacan, Bahia, Brazil | 14°47'56.8''S, 39°10'16.4''W | Fungus garden of *Atta cephalotes* | BMSR120703-01(FL5) | KM817049 | MH715127 | KM817119 | Meirelles et al. (2015b) |
| *Escovopsis* sp. | LESF 844 | BA005 | Camacan, Bahia, Brazil | 14°47'56.8''S, 39°10'16.4''W | Fungus garden of *Atta cephalotes* | BMSR120703-01(FL5) | KM817050 | MH715128 | KM817120 | Meirelles et al. (2015b) |
| *Escovopsioides nivea* | CBS 135749^ET^ | AUJ6 | Viçosa, Minas Gerais, Brazil | 20°44'31.71''S, 42°52'43.83''W | Fungus garden of *Acromyrmex subterraneus subterraneus* | --- | JQ815078 | JQ855716 | JQ855713 | Augustin et al. (2013) |
| *Hypomyces protrusum* | TFC 201316 | --- | Madagascar | --- | Eucalyptus forest | --- | FN859414 | FN859414 | FN868732 | Põldmaa (2011) |
| *Hypomyces semicirculare* | CBS 705. 88 | --- | Cuba | --- | On old  polypore | --- | NR_121425 | FN859417 | FN868735 | Põldmaa (2011) |
| *Hypomyces asterophorum* | CBS 676.77 | --- | Japan | --- | --- | --- | FN859395 | AJ583469 | FN868712 | Põldmaa (2011) |
| *Hypomyces samuelsii* | TFC 2007-23 | --- | Peru | --- | on  basidioma of an agaricoid basidiomycete on a stem of a palm | --- | FN859451 | FN859451 | FN868769 | Põldmaa (2011) |
| *Hypomyces samuelsii* | C.L.L. 7259 | --- | West Indies | --- | on *Auricularia* cf. *polytricha* on bark of Cyathea | --- | FN859445 | FN859445 | FN868764 | Põldmaa (2011) |
| *Trichoderma harzianum* | CBS 226.95 | --- | England | --- | --- | --- | AY605713 | HM466680 | AF534621 | Chaverri et al. (2003) |
| *Protocrea pallida* | TFC 99-209 | --- | New York, Cleaveland | --- | --- | --- | NR_111329 | EU710769 | EU703903 | Jaklitsch and Samuels (2011) |
| *Sphaerostilbella aureonitens* | GJS 74-87 | --- | --- | --- | --- | --- | FJ442633 | HM466683 | FJ467644 | unpublished |
| *Lecanicillium antillanum* | CBS 350.85 | --- | Cuba | --- | on  basidioma of an agaricoid | --- | NR_111097 | AF339536 | DQ522350 | Spatafora et al. (2007) |

^ET^ Ex-type.

Table S2. Molecular markers, primers and PCR conditions used in this study

| **Marker** | **Primers** | **Conditions** | **Reference** |
| --- | --- | --- | --- |
| ITS | ITS4  (5’TCCTCCGCTTATTGATATGC3’)  ITS5 (5’GGAAGTAAAAGTCGTAACAAGG3’) | 96°C for 3 min, 35 cycles at 94°C for 1 min, 55°C for 1 min and a final  extension step at 72°C for 2 min | White et al. (1990),  Schoch et al. (2012) |
| *tef1* | EF6–20F (5’AAGAACATGATCACTGGTACCT3’)  EF6–1000R (5’CGCATGTCRCGGACGGC3’) | 96°C for 3 min, 35 cycles at 96°C for 30 s, 61°C for 45 s and a final  extension step at 72°C for 1 min | Taerum et al. (2007) |
| LSU | CLA-F (5’GCATATCAATAAGCGGAGGA3’)  CLA-R (5’GACTCCTTGGTCCGTGTTTCA3’) | 96°C for 3 min, 35 cycles at 94°C for 1 min, 55°C for 1 min and a final  extension step at 72°C for 2 min | White et al. (1990), Haugland and Heckman (1998), Currie et al. (2003) |

**References of supplementary material**

Augustin JO, Groenewald JZ, Nascimento RJ, Mizubuti ESG, Barreto RW, Elliot SL, Evans HC (2013) Yet more “weeds” in the garden: Fungal novelties from nests of leaf-cutting ants. PLoS One 8 (12): e82265. https://doi.org/10.1371/journal.pone.0082265

Chaverri P, Castlebury LA, Samuels GJ, Geiser DM (2003) Multilocus phylogenetic structure within the *Trichoderma harzianum* / Hypocrea lixii complex. Mol Phylogenet Evol 27(2):302-313. https://doi: 10.1016/S1055-7903(02)00400-1

Haugland RL, Heckman JL (1998) Identification of putative sequence specific PCR primers for detection of the toxigenic fungal species *Stachybotrys chartarum*. Molecular and Cellular Probes 12(6): 387-396. https://doi.org/10.1006/mcpr.1998.0197

Jaklitsch WM, Põldmaa K, Samuels GJ (2011) Reconsideration of Protocrea ( Hypocreales , Hypocreaceae ). Mycologia 100(6): 962-984.

Masiulionis VE, Cabello MN, Seifert KA, Rodrigues A, Pagnocca FC (2015) Escovopsis trichodermoides sp. nov., isolated from a nest of the lower attine ant Mycocepurus goeldii. Antonie van Leeuwenhoek 107(3): 731-40. https://doi: 10.1007/s10482-014-0367-1

Meirelles LA, Montoya QV, Solomon SE, Rodrigues A (2015a) New light on the systematics of fungi associated with attine ant gardens and the description of *Escovopsis kreiselii* sp. nov. PLoS One 10(1): e0112067. https://doi: 10.1371/journal.pone.0112067

Meirelles LA, Solomon SE, Bacci M, Wright AM, Mueller UG, Rodrigues A (2015b) Shared Escovopsis parasites between leaf-cutting and non-leaf-cutting ants in the higher attine fungus-growing ant symbiosis. R Soc Open Sci 2(9):150257. https://doi: 10.1098/rsos.150257

Põldmaa K (2011) Tropical species of *Cladobotryum* and *Hypomyces* producing red pigments. Stud Mycol 68: 1-34. https://doi: 10.3114/sim.2011.68.01

Seifert KA, Samson RA, Chapela IH (1995) Escovopsis aspergilloides , a Rediscovered Hyphomycete from Leaf-Cutting Ant Nests. Mycologia 87(3): 407-413. https://doi:10.2307/3760838

Spatafora JW, Sung GH, Sung JM, Hywel-Jones NL, White JF Jr (2007) Phylogenetic evidence for an animal pathogen origin of ergot and the grass endophytes. Mol Ecol 16(8):1701-1711. https://doi: 10.1111/j.1365-294X.2007.03225.x

White TJ, Bruns T, Lee SH, Taylor JW (1990) PCR protocols: a guide to methods and application. Academic Press 315−322. http://dx.doi.org/10.1016/b978-0-12-372180-8.50042-1
